# Elections—University of Louisville

**Published:** 1881-11

**Authors:** 


PROF. THEOPHILUS PARVIN, of Indianapolis, has been elected to the Chair of Obstetrics in the University of Louisville, made vacant by the death of Prof. John E. Crowe.

